# Embracing Artificial Intelligence in Cardiovascular Disease Fellowship

**DOI:** 10.1016/j.jacadv.2025.101709

**Published:** 2025-04-25

**Authors:** Evan Derector, Daniel Ricketti

**Affiliations:** a Cooper Medical School of Rowan University, Camden, New Jersey, USA; b Department of Cardiovascular Disease, Cooper University Healthcare, Camden, New Jersey, USA

**Keywords:** artificial intelligence (AI), cardiovascular disease, education, fellowship, training

As artificial intelligence (AI) becomes increasingly accessible, its integration into the health care field will continue to expand. Previous research has identified the growing role of AI in cardiology as a tool to improve diagnostic accuracy, enhance patient care, increase provider efficiency, and develop more precise procedural tools to treat patients.^1^^,^^2^ With the expectation of continued exponential growth of AI, AI education should be integrated into cardiovascular disease training.

While AI has been around for over 70 years, the development of more user-friendly human-like interfaces, such as ChatGPT, has expanded the integration of AI into health care. As these tools continue to develop, centers for higher education and national organizations, such as the American Medical Association, Accreditation Council for Graduate Medical Education, and *Journal of the American College of Cardiology*, are exploring ways to educate and counsel physicians to embrace the benefits of AI.^3^ While cited for its wide ranging benefits of enhancing human capabilities, improving efficiency, and reducing costs, there is the potential for misuse, demanding the need for physicians to have educational tools to critically appraise its utilization in medical decision-making.

In 2018, an article titled *Artificial Intelligence for FITs (Fellows in Training)*, identified the need for trainees to be prepared for AI’s integration into health care, a pressure that has only increased. While AI historically was perceived as a replacement for physicians, it is now embraced to “complement what physicians do.”^3^ Dr Oscar Marroquin, MD, FACC, expresses the need for FITs, “to learn more about the basics of AI to know when to use it […] or how to interpret studies that use these techniques, as they will keep coming and improving.”^3^ Physicians and researchers continue to expand the capabilities of AI to improve physician processes and decision-making, however, not much has changed in the training available to fellows.

In 2018, the American Medical Association adopted a policy surrounding the integration of “Augmented Intelligence in Health Care” with the goal to “promote greater understanding of the promise and limitations of health care AI.”^4^ Yet, there is no standardization or promotion of a nationwide curriculum for medical trainees and practicing physicians to fulfill the potential of AI in improving efficiency and patient care. Recently, Jain et al^5^ identified the lack of appropriate training, regulations, evaluation methods, and infrastructure as major obstacles to realizing the potential of AI in the field of cardiology.^5^ However, the authors suggested that a globalized effort will help to evaluate AI’s potential enhancements to the field and “ensure AI tools and associated AI-assisted clinical workflows are validated, ethical, and effective for patient care.”^5^ Ultimately, this effort starts with our physicians of tomorrow, our FITs.

With AI’s ability to complement the role of physicians, more trainees should embrace the integration of AI into patient care and research endeavors. As Debbie Teoderescu, MD, wrote, “AI could augment the natural intelligence ‘between the eartips’” of the stethoscope.^6^ A core tenet of fellowship training is to prepare fellows for rewarding and successful careers. Therefore, it is necessary to appropriately train fellows on how to critically evaluate the role of AI in clinical decision-making and treatment of cardiovascular diseases. The Accreditation Council for Graduate Medical Education develops educational components and core competencies including, but not limited to, professionalism, patient care and procedural skills, medical knowledge, and practice-based learning; all with the goal of developing a fellow that can be “a trusted physician [that can] enter autonomous practice.”^7^ With the rapid growth of technology, graduating fellows are lacking the training and knowledge to apply technology, and more specifically, AI, to their practice. Therefore, training in AI needs to become standardized and integrated as a core competency in fellowships nationwide.

Various frameworks have been proposed for the integration of AI into medical training. One specific framework focuses on training physicians to effectively use AI technology in their practice while also being trained to recognize “its shortfalls such as transparency and liability.”^6^ However, until there is a broader understanding of how AI works and regulatory bodies to govern its uses, health care providers will be unable to tap into the potential to improve their practice and patient outcomes. To overcome these barriers, the framework proposes a wide array of AI integration in education to increase familiarity with the available tools and technologies.^6^ For example, fellows would be taught how to utilize AI to manage the electronic health record and to succinctly collect recommendations from an array of sources for decision-making in patient care, with AI-guided processes complementing the user’s medical knowledge.^6^ This framework aims to teach trainees to apply and appraise AI tools in early medical education with advancements in technology integrated into CME. Along the journey of medical training, trainees will develop their clinical reasoning skills and medical knowledge with the ability to augment their learning with AI tools and applications, simultaneously. As they narrow their focus on their desired area of practice, the tools and applications that are pertinent to their educational stage and chosen field will narrow as well. But, with the educational foundation developed early on and continued throughout training, AI will be an invaluable tool that can be applied to everyday practice.

Another framework identifies 3 fundamental ways for AI to be integrated into the current fellowship training system. This framework discusses “direct teaching,” to provide knowledge directly to the trainee; “support teaching,” as a tool to collaborate while working in a team setting; and “empowering the learner,” where fellows can work through medical concepts while using AI as a feedback tool.^8^ Each of these 3 AI processes should be embraced by fellows and integrated into training to aid in mastering the growing knowledge base required to be a successful and autonomous physician.^8^

The Artificial Intelligence Fellowship in Cardiovascular Disease at Northwestern University Feinberg School of Medicine serves as a pioneer in the field of AI education and training in cardiology, although requiring an extra year of medical training. As part of the extra year of education, AI fellows are provided with skills to utilize AI and machine learning to innovate and advance our knowledge and use of AI in treating cardiovascular diseases.^9^^,^^10^ Early advances produced in this program include computer algorithms to improve electrocardiogram diagnostics and integrated technology to interpret cardiac nuclear imaging.^9^ While trainees graduating from this program are at the forefront of cutting edge research surrounding the integration of AI into treating cardiovascular disease, the base level of knowledge required to effectively understand and appraise these tools must be integrated into all fellowship programs. If not, current general cardiology fellows, the cardiologists of tomorrow, will not have the opportunity to adapt to the changing landscape of medicine and employ tools available to provide their patients with the best care.

By more broadly accepting the integration of AI into fellowship training, universally, cardiology fellows will be better prepared to adapt to growing pressures to adopt AI, in their practice, to make more efficient and accurate decisions, while reducing health care costs. In embracing AI in our educational system now, we will better prepare the cardiologists of tomorrow to navigate the integration of AI into the health care system and develop a familiarity with the AI tools available in their own practice.

## Funding support and author disclosures

The authors have reported that they have no relationships relevant to the contents of this paper to disclose.
